# The ICD-11 Personality Disorder Trait Model Fits the Kurdish Population Better Than the DSM-5 Trait Model

**DOI:** 10.3389/fpsyt.2021.635813

**Published:** 2021-03-30

**Authors:** Azad Hemmati, Fateh Rahmani, Bo Bach

**Affiliations:** ^1^Department of Psychology, University of Kurdistan, Sanandaj, Iran; ^2^Psychiatric Research Unit, Center for Personality Disorder Research, Region Zealand Psychiatry, Slagelse, Denmark

**Keywords:** ICD-11, DSM-5, alternative model of personality disorders (AMPD), PID-5, personality disorder, personality trait, Anankastia, compulsivity

## Abstract

The ICD-11 Classification of Personality Disorders and the DSM-5 Alternative Model of Personality Disorders (AMPD) operate with trait domains that contribute to the individual expression of personality disturbance (i.e., negative affectivity, detachment, dissociality, disinhibition, anankastia, and psychoticism). To date, these trait frameworks have not been investigated sufficiently in Middle Eastern cultures. Thus, the present study explored the structure of the ICD-11 and AMPD personality disorder (PD) trait domains in a large mixed sample from the Kurdistan zone of Iran. The ICD-11 and AMPD trait domains were operationalized using empirically supported algorithms for the Personality Inventory for DSM-5 (PID-5). The PID-5 was administered to a large mixed sample (*N* = 3,196) composed of 2,678 community and 518 clinical participants. Structural validity was investigated using Exploratory Factor Analysis (EFA), whereas differential construct validity was explored by comparing clinical and community scores. Model fit and the expected factor structure were deemed appropriate for the ICD-11 trait model, but less adequate for the DSM-5 trait model (i.e., disinhibition did not emerge as a separate factor). All domain and facet scores showed significant differences between clinical and community subsamples with moderate to large effects, mostly for disinhibition and dissociality/antagonism while least for anankastia. The findings of the present study may suggest that the ICD-11 trait model is more cross-culturally fitting than the DSM-5 AMPD trait model, at least with respect to a large mixed sample from the region of Kurdistan. Accordingly, there is evidence for using PID-5 data for WHO ICD-11 purposes in this part of the World.

## Introduction

A paradigm shift has occurred in response to 30 years of demonstrated shortcomings of the categorical conceptualization of personality disorders (PD) (1, 2). Compelling evidence suggests that personality pathology is best measured using a global dimension of dysfunction, whereas specific trait dimensions may serve as sound indicators of individual, stylistic expressions of the dysfunction (3). In 2013, a dimensional and trait-focused approach to PDs was introduced in the DSM-5 Alternative Model for Personality Disorders (AMPD) (4). The AMPD model conceptualizes PDs based on a combination of overall personality functioning and specific pathological personality traits (i.e., Negative affectivity, Detachment, Antagonism, Disinhibition, and Psychoticism). Likewise, the 11th Revision of WHO's International Classification of Diseases (ICD-11) (5) includes a classification of PDs based on severity (i.e., Mild, Moderate, Severe), and allows the clinician to characterize individual expressions of personality dysfunction by means of trait domain qualifiers (i.e., Negative Affectivity, Detachment, Dissociality, Disinhibition, and Anankastia). The DSM-5 AMPD and the ICD-11 approaches are similar in many respects. However, the ICD-11 trait model differs from its DSM-5 counterpart by its inclusion of a separate Anankastia domain and exclusion of a Psychoticism domain.^1^ Research on these new trait models have provided broad support (1, 6–8), with particular emphasis on the assessment of the trait domains (9–12). More recently, a number of studies on the ICD-11 trait domains have been conducted (13–20). Nevertheless, only little research has been dedicated to these new trait models in non-Western cultures, and only few international studies have focused on the ICD-11 PD approach, despite the fact that this framework must be used for coding purposes by all WHO-member countries. For example, one Iranian study largely replicated the ICD-11 trait model in a non-clinical sample (21) using a small sample size, which may be considered insufficient and therefore warrants replication in a larger sample. The same applies to a study solely based on university students from Algeria (22). Beyond the aforementioned studies, only the AMPD trait model has been investigated in Middle Eastern cultures with some empirical support (23–25).

Given the overlap between ICD-11 and AMPD trait models and the need for more focus on WHO's international ICD-11 approach, Bach and colleagues (26) developed an algorithm that involved 16 designated PID-5 facets to assess the five proposed ICD-11 trait domains. This PID-5 operationalization of ICD-11 trait domains has subsequently been supported in several studies (21, 27, 28). For instance, Bach et al. (27) found that the PID-5-derived ICD-11 trait domains showed expected associations with categorical PD diagnoses. More recently, Sellbom et al. (29) updated Bach et al.'s (26) ICD-11 scoring algorithm by adding the PID-5 trait facets of Suspiciousness and Attention Seeking to account for more nuances of Negative Affectivity and Dissociality. This revised 18-facet scoring algorithm was validated (i.e., structural and criterion validity) using a large Canadian psychiatric inpatient sample. Thus, it should now be viable for researchers and clinicians across the world to measure the ICD-11 trait domains while simultaneously measuring DSM-5 AMPD traits.

The present study aimed to investigate and compare the psychometric features of the DSM-5 AMPD and ICD-11 trait domain operationalizations including their empirical suitability for the cultural setting of the Kurdistan region. We specifically evaluated the expected five-factor structures and the ability of AMPD and ICD-11 trait domains to distinguish between clinical and non-clinical groups.

## Method

### Procedures and Participants

The current study was based on a mixed sample of 3,196 individuals including 2,678 community participants and 518 psychiatric patients. A total of 72 cases were excluded due to incomplete responses. To ensure valid responses, we employed the PID-5 Response Inconsistency Scale (PID-5-RIS) to detect and exclude cases with random responding based on a PID-5-RIS score of 17 or above (30), which resulted in the exclusion of 301 cases. Consequently, the analyses in the present study were ultimately based on a final sample (*N* = 2,823) composed of 2,447 community participants and 376 clinical participants. All participants were native Kurds mostly residing in the western provinces of Iran. Their age ranged from 14 to 88 years (mean = 27.5; SD = 9.43) and 50.4% were woman. Data were collected in the period from 2016 until 2019.

Clinical participants were recruited from hospital settings. Community participants were voluntarily recruited via public announcements among college students.

The inclusion criteria for both groups were at least eighth-grade education along with the ability to read and speak Farsi fluently. The exclusion criteria for the community sample were a history of mental disorder, substance use, and serious medical conditions. All participants voluntarily gave their informed consent to participate, and the study was approved by a local ethical committee.

### Measure

The Personality Inventory for DSM-5 (PID-5; 23) is a 220-item self-report inventory with a four-point Likert scale ranging from 0 (very false or often false) to 3 (very true or often true). The PID-5 was constructed to measure 25 trait facet scales and five higher-order domain scores. The PID-5 was adapted to the Persian language and the Kurdish population under the supervision of its developer. We used the official APA scoring algorithm (23) to operationalize the DSM-5 AMPD trait domains, whereas the empirically supported ICD-11 scoring algorithm (26, 29) was employed to operationalize the ICD-11 trait domains.

### Statistical Approach

In order to be methodologically consistent with the initial PID-5 construction study (31), we performed exploratory factor analysis (EFA) with CF-Equamax rotation and robust maximum likelihood estimation in Mplus 7.4. Model fit was evaluated using chi-square test (χ2), root mean square error of approximation (RMSEA), comparative fit index (CFI), Tucker–Lewis index (TLI), and standardized root mean square residual (SRMR). We relied on the CFI (above 0.90) and the RMSEA (below 0.08) as indicators of adequate model fit (32, 33). Tucker's congruence coefficients (34) were calculated to compare factor loadings of the obtained DSM-5 and ICD-11 trait structures with patterns in their respective U.S. and Canadian construction studies. Group differences for computed facet and domain scores were calculated using Cohen's d effect sizes (35), where differences may be interpreted as small (0.20), medium (0.50), and large (0.80).

## Results

Socio-demographics and clinical characteristics are reported in Table 1.

**Table 1:** Socio-demographic and diagnostic characteristics of participants.

	**College sample**	**Patient sample**
	**(*n* = 2,444)**	**(*n* = 376)**
**Age range (*M*; *SD*)**	14–58 years (27.17; 9.93)	18–55 years (29.38; 7.57)
**Gender**		
Female	52.2%	30.1%
Male	46.5%	69.7%
Unidentified	1.3%	0.2%
**Marriage**		
Single	76.4%	62.5%
Married	20.4%	34.7%
Unidentified	3.2%	2.7%
**Education**		
≤High school	3.1%	34.9%
High school	15.3%	41.5%
Undergraduate students	51.6%	14.7%
Master's students	17.7%	2.1%
Doctoral students	1.6%	1.0%
Unidentified	10.5%	5.8%
**Personality disorders**		
Paranoid	-	2.3%
Schizoid	-	0.8%
Schizotypal	-	1.4%
Antisocial	-	5.0%
Borderline	-	57.3%
Histrionic	-	1.0%
Narcissistic	-	0.8%
Avoidant	-	0.8%
Dependent	-	0.2%
Obsessive-compulsive	-	1.2%
**Other mental disorders**		
Bipolar disorder, type I	-	11.2%
Major depression	-	8.9%
OCD	-	5.0%
Generalized anxiety disorder	-	1.2%
Social anxiety disorder	-	0.8%
Panic disorder	-	0.6%
Anorexia nervosa	-	0.4%
Somatic symptom disorder	-	0.6%
Body dysmorphic disorder	-	0.2%
Alcohol use disorder	-	0.4%
PTSD	-	0.2%

### Scale Reliabilities

As presented in Table 2, the alpha coefficients were satisfactory (α > 0.70) for 22 out of 25 trait facet scores, which is largely consistent with findings in other international studies. Only the facets of Suspiciousness (0.57), Submissiveness (0.59), and Intimacy avoidance (0.66) showed less adequate internal consistency. The median alpha coefficient across all facet scales was 0.81.

**Table 2:** Scale statistics and group differences for PID-5 facets and DSM-5 and ICD-11 domain scores.

	**Total**			**Community**		**Patients**		**Diff. effect**
	**(N = 2,823)**			**(n = 2,444)**		**(n = 376)**		
**Trait facets**	***M***	***SD***	**α**	***M***	***SD***	***M***	***SD***	***d***
Emotional lability	1.27	0.63	0.81	1.21	0.60	1.66	0.69	0.70
Anxiousness	1.26	0.63	0.85	1.21	0.61	1.62	0.64	0.66
Separation insecurity	1.09	0.67	0.82	1.01	0.62	1.61	0.75	0.87
Submissiveness	1.22	0.58	0.59	1.19	0.56	1.46	0.64	0.45
Perseveration	1.18	0.55	0.81	1.12	0.52	1.54	0.61	0.74
Suspiciousness	1.28	0.48	0.57	1.21	0.45	1.68	0.52	0.97
Depressivity	0.92	0.66	0.92	0.82	0.60	1.56	0.71	1.12
Withdrawal	0.97	0.60	0.87	0.90	0.55	1.41	0.68	0.82
Restricted affectivity	1.08	0.53	0.70	1.02	0.50	1.43	0.59	0.75
Intimacy avoidance	1.04	0.56	0.66	1.00	0.54	1.25	0.63	0.43
Anhedonia	1.14	0.56	0.77	1.07	0.53	1.58	0.57	0.93
Manipulativeness	0.89	0.60	0.72	0.81	0.52	1.44	0.79	0.94
Deceitfulness	0.97	0.58	0.83	0.89	0.51	1.47	0.75	0.90
Grandiosity	1.15	0.60	0.78	1.11	0.57	1.43	0.72	0.49
Hostility	1.26	0.63	0.86	1.17	0.58	1.82	0.70	1.01
Callousness	0.82	0.54	0.77	0.72	0.43	1.44	0.73	1.20
Attention seeking	1.31	0.67	0.87	1.26	0.64	1.63	0.75	0.53
Impulsivity	1.06	0.69	0.83	0.96	0.63	1.65	0.79	0.97
Irresponsibility	0.93	0.57	0.76	0.83	0.49	1.55	0.69	1.20
Distractibility	1.15	0.64	0.88	1.06	0.60	1.70	0.64	1.03
Rigid perfectionism	1.38	0.54	0.80	1.37	0.53	1.46	0.57	0.16
Risk taking	1.42	0.44	0.75	1.38	0.42	1.65	0.54	0.56
Eccentricity	0.95	0.68	0.94	0.88	0.64	1.43	0.73	0.80
Perceptual dysregulation	0.88	0.58	0.87	0.80	0.51	1.38	0.70	0.95
Unusual beliefs	0.91	0.61	0.83	0.84	0.56	1.30	0.75	0.70
**DSM-5 Domains**								
DSM-5 Negative Affectivity	1.21	0.55		1.14	0.51	1.63	0.58	0.86
DSM-5 Detachment	1.05	0.47		0.99	0.44	1.41	0.51	0.88
DSM-5 Antagonism	1.00	0.51		0.94	0.44	1.45	0.67	0.90
DSM-5 Disinhibition	1.04	0.56		0.95	0.49	1.63	0.62	1.22
DSM-5 Psychoticism	0.91	0.56		0.84	0.51	1.37	0.68	0.88
**ICD-11 Domains**								
ICD-11 Negative affectivity	1.18	0.51		1.11	0.46	1.63	0.54	1.04
ICD-11 Detachment	1.03	0.47		0.98	0.43	1.36	0.54	0.72
ICD-11 Dissociality	1.09	0.49		1.01	0.42	1.55	0.63	1.01
ICD-11 Disinhibition	1.14	0.47		1.06	0.41	1.64	0.56	1.18
ICD-11 Anankastia	1.28	0.49		1.24	0.47	1.50	0.54	0.51

*N = 2,823. α = alpha coefficients*.

*All group differences were statistically significant below the 0.001 level, except for Rigid Perfectionism, which was significant at the 0.002 level*.

### Replication of the Five-Factor Structure for DSM-5 and ICD-11 Models

As presented in Tables 3, 4, the EFA analyses yielded five higher-order factors for both the DSM-5 and the ICD-11 models. The ICD-11 model largely showed the expected pattern, whereas the DSM-5 model only partially aligned with the expected pattern, as it did not yield a separate factor of Disinhibition. Instead, a separate domain with predominant features of Compulsivity/Anankastia emerged in the DSM-5 model, whereas features of Disinhibition were intermingled with Negative Affectivity.

**Table 3:** DSM-5 five-factor loadings, factor correlations, and congruence coefficients.

	**F1**	**F2**	**F3**	**F4**	**F5**
Depressivity ^NA,DT^	**0.59**	0.32	0.07	0.02	0.18
Anxiousness ^NA*^	**0.56**	0.10	−0.13	0.33	0.14
Distractibiliy ^DI*^	**0.55**	0.16	0.13	0.14	0.16
Emotional lability ^NA*^	**0.44**	−0.11	0.09	0.37	0.25
Impulsivity ^DI*^	**0.44**	0.03	0.34	0.00	0.19
Separation insecurity ^NA*^	0.37	−0.13	0.21	0.31	0.13
Withdrawal ^DT*^	0.09	**0.67**	0.04	0.12	0.11
Restricted affectivity ^DT,−NA^	−0.08	**0.64**	0.15	0.15	0.09
Intimacy avoidance ^DT*^	−0.12	**0.63**	−0.05	0.00	0.11
Anhedonia ^DT*^	**0.44**	**0.54**	0.01	0.02	0.03
Manipulativeness ^AG*^	−0.10	0.05	**0.67**	0.12	0.19
Callousness ^AG^	−0.02	0.36	**0.64**	−0.02	0.17
Deceitfulness ^AG*^	0.15	0.04	**0.61**	0.08	0.23
Irresponsibility ^DI*^	0.35	0.20	**0.49**	−0.13	0.19
Hostility ^NA,AG^	0.26	0.17	**0.45**	0.32	−0.05
Risk taking ^DI^	−0.07	−0.09	0.39	0.00	0.26
Rigid perfectionism ^−DI^	−0.11	0.19	−0.18	**0.77**	0.11
Attention seeking ^AG^	0.12	−0.20	0.39	**0.51**	0.08
Grandiosity ^AG*^	−0.25	0.04	0.34	**0.46**	0.22
Perseveration ^NA^	0.31	0.17	−0.01	**0.44**	0.22
Submissiveness ^NA^	0.28	0.02	0.05	0.38	0.06
Suspiciousness ^NA,DT^	0.17	0.16	0.20	0.25	0.17
Unusual beliefs ^PS*^	−0.12	0.03	−0.01	0.08	**0.87**
Perceptual dysregulation ^PS*^	0.24	0.10	0.06	0.06	**0.67**
Eccentricity ^PS*^	0.07	0.19	0.07	0.07	**0.60**
**Factor correlations**					
F2	0.33				
F3	0.31	0.27			
F4	0.35	0.26	0.30		
F5	0.40	0.49	0.53	0.55	
**Tucker's congruence**					
U.S. construction study (31)	0.76	0.75	0.83	−0.02	0.95

*N = 2,823*.

*NA, Negative Affectivity; DT, Detachment; AG, Antagonism; DI, Disinhibition; PS, Psychoticism; Loadings above 0.40 are boldfaced*.

**Table 4:** ICD-11 five-factor loadings, factor correlations, and congruence coefficients.

	**F1**	**F2**	**F3**	**F4**	**F5**
Anxiousness ^NA^	**0.92**	−0.02	−0.04	−0.02	0.07
Depressivity ^NA^	**0.54**	0.25	0.06	0.31	−0.09
Suspiciousness ^NA^	**0.42**	0.10	0.30	0.00	0.15
Intimacy avoidance ^DT^	−0.02	**0.67**	−0.03	−0.03	0.06
Withdrawal ^DT^	0.18	**0.65**	0.04	0.09	0.11
Restricted affectivity ^DT^	0.02	**0.62**	0.15	0.05	0.18
Manipulativeness ^DT^	0.09	0.06	**0.69**	0.04	0.11
Callousness ^DS^	0.03	0.36	**0.63**	0.18	−0.01
Grandiosity ^DS^	−0.05	0.05	**0.44**	−0.02	**0.52**
Risk taking ^DI^	−0.07	−0.10	**0.44**	0.18	0.11
Hostility ^DS^	0.26	0.05	0.35	0.25	0.18
Impulsivity ^DI^	0.07	0.02	0.17	**0.66**	0.01
Distractibility ^DI^	0.21	0.15	−0.07	**0.65**	0.11
Irresponsibility ^DI^	0.13	0.21	0.36	**0.49**	−0.14
Emotional lability ^NA^	0.29	−0.12	0.03	**0.44**	0.34
Rigid perfectionism ^AK^	0.12	0.18	−0.10	−0.08	**0.75**
Perseveration ^AK^	0.23	0.18	−0.08	0.36	**0.44**
Attention seeking ^DS^	0.10	−0.22	0.33	0.23	**0.44**
**Factor correlations**					
F2	0.37				
F3	0.24	0.29			
F4	0.62	0.34	0.48		
F5	0.48	0.23	0.31	0.31	
**Tucker's congruence**					
Canadian patients (29)	0.84	0.87	0.82	0.84	0.68

*N = 2,823. NA, Negative Affectivity; DT, Detachment; DS, Dissociality; DI, Disinhibition; AK, Anankastia*.

*Loadings above 0.40 are boldfaced*.

The fit indices were acceptable for both the DSM-5 five-factor model [χ^2^ = 2049.48 (*df* = 185); RMSEA = 0.060; CFI = 0.955; TLI = 0.927; SRMR = 0.021] and the ICD-11 five-factor model [χ^2^ = 532.83 (*df* = 73); RMSEA = 0.047; CFI = 0.982; TLI = 0.963; SRMR = 0.014].

We used Tucker's formula to estimate congruence coefficients with the original construction studies. With respect to the DSM-5 model, four out of the five extracted factors showed some congruence with the U.S. construction study (23), with a total mean congruence coefficient of 65. For the ICD-11 model, all the five extracted factors showed some congruence with the related Canadian construction study (19), with a mean congruence coefficient of 81.

### Facet and Domain Level Group Differences

The differential construct validity of DSM-5 and ICD-11 trait models was investigated by means of independent *t*-test and Cohen's d effect size for differences between clinical and community samples.

With respect to PID-5 trait facet scores, all group differences were statistically significant at the 0.001 level, except for Rigid Perfectionism, which was significant at the 0.002 level. The majority of facet scores showed group differences with medium to large effect sizes. The effect sizes generally ranged from 0.43 to 1.20, except for the facet of Rigid Perfectionism, which showed a very small differential effect size (*d* = 0.16).

With respect to trait domain scores, both the DSM-5 and ICD-11 models showed statistically significant group differences. All the DSM-5 trait domains showed large effect sizes, while the ICD-11 domains showed medium to large effect sizes, which is particularly attributed to the domain of Anankastia (*d* = 0.51).

## Discussion

The present comparative study aimed at evaluating the structural and differential construct validity of the ICD-11 and DSM-5 AMPD trait models in a large mixed sample derived from the Kurdistan region of Iran. We overall found appropriate model fit for both DSM-5 and ICD-11 five-factor solutions. However, the factor loading pattern was less adequate for the DSM-5 trait model (i.e., Disinhibition did not emerge as a separate factor). All domain and facet scores significantly differentiated between clinical and community samples primarily with medium to large effect sizes. Taken together, these findings therefore suggest that the ICD-11 trait model may be somewhat more cross-culturally appropriate than the DSM-5 AMPD trait model, at least with respect to a large mixed sample from the region of Kurdistan. Accordingly, the ICD-11 PD trait domain operationalization may potentially have superior utility in this region of the World. In the following, we will further discuss certain patterns in our findings.

First, in the present study, the expected five-factor structure for the DSM-5 trait model was only partially supported. Accordingly, the factor analysis did not yield a separate factor for Disinhibition. Instead, the Disinhibition facets were included in the factors of Negative Affectivity (e.g., Impulsivity) and Antagonism (e.g., Irresponsibility). Nevertheless, this deviation remains conceptually coherent because Impulsivity is also a well-established feature of Neuroticism within the five-factor model of personality (36); it is also empirically well-established that Disinhibition and Antagonism overlap within one joint externalizing factor (37). The primary loading of Impulsivity on the Negative Affectivity domain is also consistent with previous research using Iranian (24) and Spanish-speaking (38) samples, suggesting that this pattern may be more pronounced in certain cultures. From a conceptual and clinical perspective, it also makes sense that Negative Affectivity (e.g., emotional dysregulation) co-occurs with features of Impulsivity (39, 40). In contrast to the DSM-5 Model, the ICD-11 model produced a somewhat pure Disinhibition factor, which, nevertheless, was also characterized by the facet of Emotional Lability.

Secondly, the five-factor structure of both DSM-5 and ICD-11 models yielded a distinct factor of Compulsivity/Anankastia, which aligns with the ICD-11 trait framework but not the DSM-5 framework. This is consistent with findings from EFA analysis of clinical PID-5 data from the United Arab Emirates (25), which yielded a distinct Compulsivity factor (i.e., Rigid Perfectionism and Perseveration), while facets of Disinhibition loaded on Antagonism and Negative Affectivity. Moreover, this study from United Arab Emirates did not yield a separate factor of Psychoticism. In the present study, both the ICD-11 and DSM-5 factors of Compulsivity/Anankastia included expected facets of Rigid Perfectionism and Perseveration but also unexpected facets of Grandiosity and Attention Seeking. This factorial pattern is consistent with the empirical and conceptual association between obsessive-compulsive personality features and a narcissistic sense of superiority (41).

Third, the ICD-11 trait domain of Anankastia, including the facet of Rigid Perfectionism, only showed small differential effects between clinical and community participants. This finding is not surprising because such anankastic features, including orderliness, perfectionism, and perseveration, characterize many healthy individuals with resources and goal-directedness (42). However, it still seems informative to portray stylistic features of Anankastia and perfectionism because only global PD severity determines whether and to what extent the individual's personality is actually disordered (43). For example, elevated features of Anankastia may involve appropriate self-discipline and goal-directedness in individuals with overall healthy personality functioning, whereas it may compromise cooperation and personal fulfillment in individuals with impaired personality functioning. Nevertheless, the small difference between clinical and community scores on the domain of Anankastia may also be attributed to cultural aspects in the Region of Kurdistan if not the particular community population of primarily college students.

### Limitations and Future Directions

The findings of the present study must be interpreted in the light of certain potential limitations along with recommendations for future research. First, in the present study we operationalized both DSM-5 and ICD-11 trait domains using the PID-5, which was originally constructed to exclusively capture the DSM-5 trait domains (31). Thus, future research should conduct a comparative evaluation of DSM-5 vs. ICD-11 trait models in Middle Eastern culture using a measure specifically developed for the ICD-11 trait domains such as the Personality Inventory for ICD-11 (PiCD) (13) or the Personality Assessment Questionnaire for ICD-11 (PAQ-11) (20). Second, the present study investigated differences between two groups that were not entirely matched in terms of age, gender, socio-demographics, and sample size. Thus, future studies using larger and better matched samples are warranted. Finally, the present study only investigated similarity with patterns of factor loadings in North American studies using Tucker's congruence coefficients (44). However, we are well-aware that this approach to investigating factorial similarity is insufficient in comparison to more stringent measurement invariance (45). We therefore recommend future research to formally investigate measurement invariance for both DSM-5 and ICD-11 trait models between Western and Middle Eastern countries, which was beyond the scope of the present study.

## Data Availability Statement

The raw data supporting the conclusions of this article will be made available by the authors, without undue reservation.

## Ethics Statement

The studies involving human participants were reviewed and approved by IR.UOK.REC.1397.016. The patients/participants provided their written informed consent to participate in this study.

## Author Contributions

BB: the supervisor, the main parts of data analysis, and proofreading. AH: data gathering, preparing the manuscript, and some parts of data analysis. FR: data gathering and preparing the manuscript.

## Conflict of Interest

The authors declare that the research was conducted in the absence of any commercial or financial relationships that could be construed as a potential conflict of interest.

## Footnotes

^1^The decision not to include psychoticism as a trait domain in ICD-11 is consistent with the WHO tradition of explaining such features entirely within the psychotic spectrum disorders.
